# Nonclassical Biofilms Induced by DNA Breaks in Klebsiella pneumoniae

**DOI:** 10.1128/mSphere.00336-20

**Published:** 2020-06-10

**Authors:** Yan Liu, Chao Pan, Lijun Ye, Yue Si, Changhao Bi, Xiaoting Hua, Yunsong Yu, Li Zhu, Hengliang Wang

**Affiliations:** a State Key Laboratory of Pathogen and Biosecurity, Beijing Institute of Biotechnology, Beijing, China; b Tianjin Institute of Industrial Biotechnology, Chinese Academy of Sciences, Tianjin, China; c Department of Infectious Diseases, Sir Run Run Shaw Hospital, College of Medicine, Zhejiang University, Hangzhou, China; University of Georgia

**Keywords:** *Klebsiella pneumoniae*, biofilm, Cas9, double-strand break

## Abstract

Biofilms usually form when the density of bacteria increases during the middle to late periods of growth in culture, commonly induced by quorum-sensing systems. Biofilms attach to the surfaces of either living or nonliving objects and protect bacteria against antibiotics and a host’s immune system. Here, a novel type of biofilm (the “R-biofilm”) is reported. These biofilms were formed by clinically isolated Klebsiella pneumoniae strains following double-stranded-DNA breaks (DSBs), while undamaged bacteria did not form classic biofilms even in the later stages of growth. R-biofilms had a fixed ring-like or discoid shape with good ductility and could protect many living bacterial cells within. We show that extracellular proteins and DNAs released, probably by dead bacteria, were the core structural materials of R-biofilms. We anticipate that novel signaling pathways besides the bacterial SOS response are involved in R-biofilm formation. The observations in this study suggest a limitation to the use of the currently popular Cas9-mediated bactericidal tools to eliminate certain bacteria because the resulting DSBs may lead to the formation of these protective R-biofilms.

**IMPORTANCE** Many pathogenic bacteria can form biofilm matrices that consist of complex molecules such as polysaccharides, proteins, and DNA. These biofilms help the bacteria to infect and colonize a host. Such biofilms may attach and develop on the surfaces of indwelling medical devices or other supportive environments. This study found that following double-strand breaks in their DNA, Klebsiella pneumoniae cells can form a novel type of biofilm with ring-like or discoid morphology. This biofilm structure, named the “R-biofilm,” helps protect the bacteria against adverse conditions such as exposure to ethanol, hydrogen peroxide, and UV radiation.

## Abstract (precis)

Many pathogenic bacteria can form biofilm matrices that consist of complex molecules such as polysaccharides, proteins, and DNA. These biofilms help the bacteria to infect and colonize a host. Such biofilms may attach and develop on the surfaces of indwelling medical devices or other supportive environments. This study found that following double-strand breaks in their DNA, Klebsiella pneumoniae cells can form a novel type of biofilm with ring-like or discoid morphology. This biofilm structure, named the “R-biofilm,” helps protect the bacteria against adverse conditions such as exposure to ethanol, hydrogen peroxide, and UV radiation.

## OBSERVATION

Klebsiella pneumoniae is an opportunistic pathogen that can form biofilm matrices that consist of polysaccharides, proteins, and DNA (1, 2). These biofilms may attach and develop on the surfaces of indwelling medical devices or other supportive environments (3) and help the bacteria to colonize a host, resulting in nosocomial infections (2, 4–6). Clinically isolated strains of K. pneumoniae are usually multidrug resistant (7, 8) and can severely reduce the quality of life of patients, which is a considerable public health concern. Recently, many studies have tried to use the CRISPR-Cas9 technique to eliminate certain bacteria by use of bacteriophages or bacterial conjugation. This technique could also be used to modulate intestinal microbiota and has become a hot topic in the field of bacterial research (9, 10). The CRISPR-Cas9 technique allows targeted editing of genomes by inducing double-stranded-DNA breaks (DSBs) (11). Unlike eukaryotes, bacteria usually lack a nonhomologous end-joining repair system; therefore, if homologous recombination segments are not provided, the bacteria die as a result of the DSBs.

**FIG 1:**
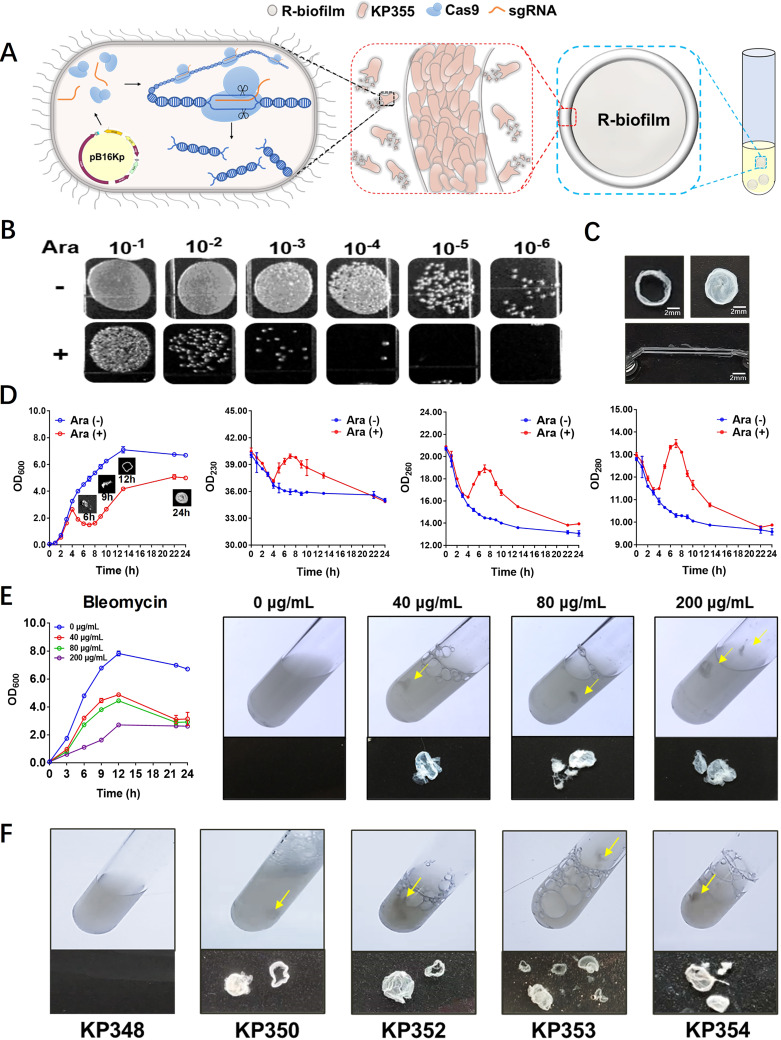
The formation of nonclassical biofilms induced by DNA breaks. (A) A CRISPR-Cas9 bactericidal plasmid (pB16Kp) derived from pBBR1MCS2 can be introduced into bacteria through conjugation or transformation to achieve targeted double-strand breaks in DNA. (B) Klebsiella pneumoniae strain 355 harboring bactericidal plasmid pB16Kp (355/pB16Kp) was induced with 0.5‰ arabinose for 6 h and counted on plates by serial dilution. The mean bacterial mortality rate was 98.74%, with a standard deviation of 0.69%. (C) Morphology, characteristics, and ductility of the ring-shaped biofilms (R-biofilms). The average diameter of the R-biofilms was approximately 5 to 6 mm after 12 h, and they exhibited good ductility, stretching to more than twice their original diameter. (D) The bacterial density (OD_600_) started to decline at 4 h and reached its lowest level at about 7 h, when 355/pB16Kp was induced by arabinose [Ara (+)]. During this period, R-biofilms began to form. After 12 h, these objects adopted a ring-like shape (and hence were named “R-biofilms”); after 24 h, the central portion of the rings filled up and a discoid shape was attained. OD_230_, OD_260_, and OD_280_ in the supernatant began to rise at 4 h and peaked at 7 h. 355/pB16Kp without arabinose was the control group [Ara (−)]. (E) Bleomycin was added to strain 355 at concentrations of 40, 80, and 200 μg/ml. After culturing at 37°C for 24 h, the growth of bacteria was inhibited at these concentrations; all the concentrations caused 355 to produce R-biofilm structures. (F) R-biofilms were also generated by 80% (4/5) of other tested clinically isolated multidrug-resistant strains of K. pneumoniae. Since some strains are resistant to kanamycin, we replaced the resistance cassette of the bactericidal plasmid with that for apramycin. The yellow arrows indicate the R-biofilms that detached from the bottom after shaking the test tube.

In this study, surprisingly, we found a previously unreported type of biofilm when we adopted the above-described strategy to kill K. pneumoniae, while the bacteria did not form classical biofilms even in the later stages of growth. We designed a CRISPR-Cas9 bactericidal plasmid (pB16Kp) with an arabinose-inducible Cas9 protein, and a guide RNA (gRNA) targeting the 16S rRNA sequence (N20-TGAAATGCGTAGAGATCTGG-) in the genome of K. pneumoniae strain 355, aiming to cause multiple fatal breaks in the cellular DNA (Fig. 1A). We constructed the plasmid using the Golden Gate cloning method and transformed it into strain 355. Strain 355 carrying the bactericidal plasmid (named 355/pB16Kp) was then cultured at 37°C with shaking (200 rpm) in 0.5‰ arabinose to induce DSBs. The control group was cultured in the absence of arabinose. Considering that the DNA break sites may be repaired by error-prone DNA polymerases using homologous recombination from another unbroken copy on the genome during replication, we first tested the bactericidal efficiency of the designed CRISPR-Cas9 system. After 6 h, a significant difference in the number of viable bacteria was observed (Fig. 1B), and the mean bacterial mortality rate was 98.74%, with a standard deviation of 0.69%.

## 

### Basic characteristics of R-biofilm.

Unexpectedly, we observed that flocculent deposits formed in the culture tubes containing arabinose after 6 h of induction, and these objects adopted a ring-like shape after 12 h of incubation. They were thus named “R-biofilms” (“R” for ring). The central portion of the rings gradually filled up, and a discoid shape was attained after 24 h. The R-biofilms were usually found as precipitates at the bottoms of the tubes and suspended in the liquid phase after shaking. The average diameter of the R-biofilms was approximately 5 to 6 mm after 12 h of growth, and they exhibited good ductility, stretching to more than twice their original diameter (Fig. 1C). In all scenarios in which controls (without the N20 sequence) or wild-type 355 was cultured, R-biofilms did not appear (see Fig. S1 in the supplemental material). To further study the relationship between R-biofilm formation and bacterial growth, we detected the optical density of the culture at 600 nm (OD_600_), OD_230_, OD_260_, and OD_280_ in the supernatant at different times. OD_230_, OD_260_, and OD_280_ are used as spectrophotometric measurements of carbohydrate, etc., oligonucleotides, and proteins, respectively. The results (Fig. 1D) showed that when 355/pB16Kp was induced by arabinose, the bacterial density (OD_600_) started to decline at 4 h and reached its lowest level at about 7 h. During this period, R-biofilms began to form. Correspondingly, OD_230_, OD_260_, and OD_280_ in the supernatant began to rise at 4 h and peaked at 7 h. These results indicated that DSB-induced cell death resulted in the release of some intracellular molecules, such as proteins, nucleic acids, and sugars, to the supernatant, which may have stimulated the R-biofilm formation.

10.1128/mSphere.00336-20.2 FIG S1 Formation of R-biofilm in K. pneumoniae 355. Wild-type K. pneumoniae 355 (355), 355 containing pB16Kp (355/pB16Kp), and 355 with an empty plasmid without an N20 sequence (355/pB16Kp-control) were cultured at 37°C with shaking (200 rpm) in 0.5‰ arabinose and kanamycin (50 μg/ml). R-biofilms formed only in the 355/pB16Kp group after arabinose induction. Download FIG S1, PDF file, 0.1 MB. Copyright © 2020 Liu et al. 2020 Liu et al. This content is distributed under the terms of the Creative Commons Attribution 4.0 International license.

### Universality of R-biofilms.

To determine whether other methods of triggering DNA breaks could lead to R-biofilm formation, bleomycin (12), which can induce DSBs, was added to cultures of strain 355. After culturing at 37°C, we found that the growth of bacteria was inhibited at three bleomycin concentrations, and interestingly, R-biofilms formed within 24 h (Fig. 1E). However, another type of antibiotic, ciprofloxacin (13), which causes indirect damage to DNA by interacting with the A subunit of DNA helicase to inhibit the synthesis and replication of bacterial DNA, did not lead to the formation of R-biofilms, although growth of the bacteria was also inhibited and a flocculent biofilm was observed at 24 h (Fig. S2). These results indicated that R-biofilm formation was mainly induced by severe injury to the genome, such as DSBs. To further examine the universality of this phenomenon, we transformed the bactericidal plasmid (with a different antibiotic resistance gene) into five other clinically isolated multidrug-resistant K. pneumoniae strains (strains 348, 350, 352, 353, and 354). The same ring-shaped structure was found for four of these five strains after induction (Fig. 1F). This suggests that the formation of R-biofilms induced by DNA breaks occurs commonly in K. pneumoniae.

10.1128/mSphere.00336-20.3 FIG S2 Flocculent structures were induced by ciprofloxacin in 355. Ciprofloxacin (40, 80, or 200 μg/ml) was incubated with strain 355. After culturing at 37°C for 24 h, the growth of bacteria was inhibited at each concentration, significantly so at 200 μg/ml. High concentrations (80 and 200 μg/ml) of ciprofloxacin caused 355 to produce flocculent structures. Download FIG S2, PDF file, 0.1 MB. Copyright © 2020 Liu et al. 2020 Liu et al. This content is distributed under the terms of the Creative Commons Attribution 4.0 International license.

### Possible mechanisms of R-biofilm production.

**FIG 2:**
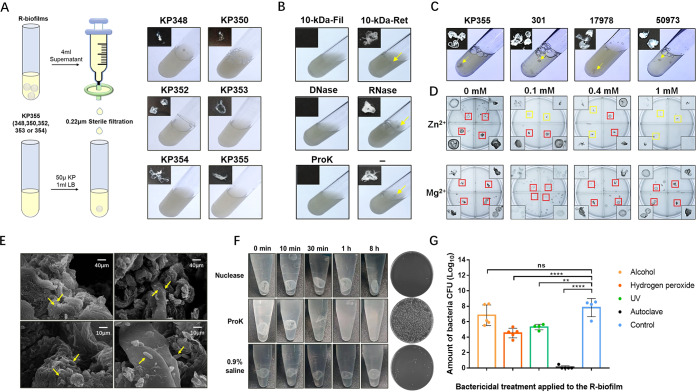
R-biofilms help protect bacteria against adverse conditions. (A) The supernatant of culture of R-biofilm-producing bacteria (strain 355/pB16Kp) was passed through a 0.22-μm filter. Then 50 μl of wild-type K. pneumoniae (strain 348, 350, 352, 353, 354 or 355) (OD_600_ = 5.0) and 1 ml of fresh LB broth were added into 4 ml of filtered supernatant. After 24 h of growth at 37°C and 200 rpm, the bacterial cultures of all of these strains formed R-biofilms. (B) The supernatant of cultures in which R-biofilms had formed was filtered using a 0.22-μm filter and then a 10-kDa cutoff filter. Additionally, DNase, RNase, and proteinase K (ProK) were respectively added to the filtrate from the 0.22-μm filter. Then 50 μl of wild-type 355 (OD_600_ = 5.0) in 1 ml of fresh LB broth was mixed with the above-treated supernatant samples and cultured at 37°C and 200 rpm for 24 h. The retentate (Ret) after filtration using the 10-kDa-cutoff membrane could stimulate the bacteria to form R-biofilms, while the low-molecular-weight filtrate (Fil) could not. The 0.22-μm filtrate after RNase digestion could stimulate the bacteria to form R-biofilms, while the 0.22-μm filtrate digested by proteinase K or DNase could not. (C) The supernatant of culture of 355/pB16Kp in which R-biofilms had formed was filtered using a 0.22-μm filter. Then we added 50 μl of wild-type Shigella flexneri 2a 301 (301) (OD_600_ = 4.0), Acinetobacter baumannii ATCC 17978 (17978) (OD_600_ = 6.5), or *Salmonella* serovar Paratyphi CMCC 50973 (50973) (OD_600_ = 4.0) to 1 ml of fresh LB broth and 4 ml of filtered supernatant and incubated the cultures for 24 h. All of these strains formed R-biofilms. (D) Zn^2+^ and Mg^2+^ (as a control) cultured with strain 355/pB16Kp and 0.5‰ arabinose (added metal ion concentrations, 0.1, 0.4, and 1 mM). Mg^2+^ had no effect on the formation of R-biofilms, while Zn^2+^ treatment caused the bacteria to produce flocculent structures. The red boxes indicate R-biofilms, and the yellow boxes indicate flocculent structures. (E) Complete 355 bacterial structures could be observed in the cross section and internal parts of the annular R-biofilm using an electron microscope. A larger photo is shown in Fig. S7. (F) R-biofilms (formed from strain 355) were incubated with solutions containing Benzonase nuclease (50 U/ml) or proteinase K (100 μg/ml); 0.9% saline was used in the control group. Proteinase K could completely disrupt the structures within 1 h; nuclease could not disrupt the structures. Proteinase lysates generated from one classic R-biofilm with a diameter of 5 mm, which was formed after 24 h of incubation in a test tube, were plated onto LB agar plates and cultured at 37°C overnight. About 1 × 10^3^ CFU of bacteria were counted after 10,000-fold dilution. That is, there were approximately 1 × 10^7^ CFU of viable bacteria in each R-biofilm. (G) R-biofilms of similar sizes were treated with different bactericidal agents, including 75% ethanol for 20 min, 10% hydrogen peroxide for 60 min, UV radiation for 5 min, and an autoclave sterilizer at 121°C for 30 min. The control group used 2.5 × 10^7^ CFU of bacteria. After treatment, the R-biofilms were injected intraperitoneally into mice. Each value represents the mean of log_10_-transformed CFU of bacteria in the livers of individual mice. Unpaired one-way analysis of variance (ANOVA) was used to evaluate differences between groups (**, *P < *0.01; ****, *P < *0.0001; ns, no statistically significant difference).

We first tested whether culture supernatant of bacteria that had formed R-biofilms could induce wild-type bacteria (without the bactericidal plasmid) to form similar R-biofilms. The culture supernatant from induced strain 355/pB16Kp (37°C for 12 h) was filtered through a 0.22-μm filter, and then 50 μl of wild-type bacteria (OD_600_ = 5.0) in 1 ml of fresh lysogeny broth (LB) was added into 4 ml of filtered supernatant and cultured at 37°C for 24 h. The results showed that substances released into the culture medium after the bacterial DNA breaks could recruit all the test K. pneumoniae strains (348, 350, 352, 353, 354, and 355) to form the same ring-shaped structures (Fig. 2A). To analyze which components are involved in the formation of R-biofilms, 4-ml quantities of culture supernatant treated by various methods were added into 1 ml of fresh LB medium containing 50 μl of strain 355 (OD_600_ = 5.0) to observe the biofilm formation. First, the culture supernatant was separated by ultrafiltration (10-kDa-molecular-weight-cutoff membrane) and the retentate and filtrate were tested. After culture at 37°C for 24 h, only the retentate could induce R-biofilm formation, indicating that some macromolecule(s) was sufficient to produce the biofilms (Fig. 2B). The 0.22-μm filtrate was used in subsequent experiments, since the 10-kDa filtrate could not stimulate the bacteria to form R-biofilms. The 0.22-μm filtrate was treated with DNase (50 μg/ml), RNase (20 μg/ml), or proteinase K (80 μg/ml). The material digested with RNase could still stimulate the bacteria to form R-biofilms, while that digested by DNase or proteinase K could not (Fig. 2B). Cocultivation of 355/pB16Kp with 0.5‰ arabinose and proteinase K, DNase, or RNase led to similar results (Fig. S3). These results indicated that extracellular DNA and proteins are involved in R-biofilm formation.

10.1128/mSphere.00336-20.4 FIG S3 Cocultivation of strain 355/pB16Kp with 0.5‰ arabinose and proteinase K, DNase, or RNase. Proteinase K, DNase, and RNase were added to a tube and cocultivated with strain 355/pB16Kp in the presence of 0.5‰ arabinose at 37°C for 24 h. Strain 355/pB16Kp formed R-biofilms in the presence of RNase but not in the presence of DNase or proteinase K. Download FIG S3, PDF file, 0.1 MB. Copyright © 2020 Liu et al. 2020 Liu et al. This content is distributed under the terms of the Creative Commons Attribution 4.0 International license.

But it was still not clear whether DNAs and proteins in the supernatant were stimulating factors or structural components of R-biofilms. Thus, we added the supernatant into cultures of other strains, including Shigella flexneri 2a strain 301, Acinetobacter baumannii ATCC 17978, and Salmonella enterica serovar Paratyphi CMCC 50973, and cultured them at 37°C for 24 h. It could be observed that similar ring-shaped biofilm structures were formed in the medium containing each of these strains (Fig. 2C). However, when the strains were cultured by adding bleomycin at an appropriate concentration to inhibit bacterial growth (Fig. S4), as we did previously for K. pneumoniae strain 355 (Fig. 1E), all of them failed to form biofilms. The above-described results are supportive of the conclusion that both DNA and proteins are structural components of R-biofilms, rather than factors that stimulate their formation. However, because of the limitations of our detection methods, we are not able to determine the role of extracellular capsular polysaccharides at this time.

10.1128/mSphere.00336-20.5 FIG S4 Inhibition of bleomycin on bacterial growth. K. pneumoniae 355, Shigella flexneri 2a 301 (301), Acinetobacter baumannii ATCC 17978 (17978), and *Salmonella* serovar Paratyphi CMCC 50973 (50973) were cultured at 37°C in the presence of 40 μg/ml, 1 μg/ml, 200 μg/ml, and 10 μg/ml of bleomycin, respectively; the different concentrations used were due to the different resistances of the different strains to bleomycin. All strains failed to form biofilms. Each value in the graph represents the mean ± standard deviation of OD_600_ in the bacterial culture medium of individual tubes from each group (*n* = 3). The unpaired *t* test was used to evaluate differences between OD_600_ values (*, *P < *0.1; **, *P < *0.01; ****, *P < *0.0001). Download FIG S4, PDF file, 0.1 MB. Copyright © 2020 Liu et al. 2020 Liu et al. This content is distributed under the terms of the Creative Commons Attribution 4.0 International license.

So, which signal pathway(s) might be involved in the formation of R-biofilms? In general, severe DNA damage in cells would lead to the SOS response. We tested this possibility first. Since zinc can inhibit the SOS response by interaction with RecA (14), 355/pB16Kp was incubated with different concentrations of Zn^2+^ and Mg^2+^ (as a control) in LB medium containing 0.5‰ arabinose. Mg^2+^ had no effect on the formation of R-biofilms, while Zn^2+^ caused the bacteria to produce a flocculent structure, suggesting that Zn^2+^ could obstruct the production of the biofilms but not completely prevent it. There are presumably other unknown mechanisms involved in the formation of R-biofilm structures together with the SOS system (Fig. 2D and Fig. S5).

10.1128/mSphere.00336-20.6 FIG S5 Effects of Zn^2+^ and Mg^2+^ on R-biofilm formation. Shown are unprocessed photographs of a 4-quadrant petri dish, with each quadrant representing one parallel test. Mg^2+^ had no effect on the formation of R-biofilms, while Zn^2+^ caused the bacteria to produce a flocculent structure. Download FIG S5, PDF file, 0.2 MB. Copyright © 2020 Liu et al. 2020 Liu et al. This content is distributed under the terms of the Creative Commons Attribution 4.0 International license.

10.1128/mSphere.00336-20.7 FIG S6 Numbers of bacteria in the livers of mice inoculated with R-biofilms at different times. (A) Images of the livers correspond to the time points in the graphs (group A, livers harvested 6 h after injection; group B, 12 h; group C, 18 h; group D, 24 h). AC to DC were controls (mice injected with saline), and numbers 1 to 3 indicate replicate samples. (B) The numbers of bacteria were slightly higher in the livers harvested at 6 h and similar between the livers harvested at the other time points. Each value represents the mean of log_10_-transformed CFU of bacteria in the livers of individual mice. Unpaired one-way ANOVA was used to evaluate differences between groups (****, *P < *0.0001). Download FIG S6, PDF file, 0.1 MB. Copyright © 2020 Liu et al. 2020 Liu et al. This content is distributed under the terms of the Creative Commons Attribution 4.0 International license.

10.1128/mSphere.00336-20.8 FIG S7 Cross section and internal parts of the R-biofilm under electron microscope. Download FIG S7, PDF file, 0.6 MB. Copyright © 2020 Liu et al. 2020 Liu et al. This content is distributed under the terms of the Creative Commons Attribution 4.0 International license.

### Bioactivity and function of R-biofilm.

An important function of biofilms is to protect bacteria against hostile environments and help the bacteria develop invasive infections within a host. To help understand the micromorphology of R-biofilms and their relationship with living bacteria, electron microscopy was performed; it revealed integrated bacterial structures on the cross sections and surface of the rings (Fig. 2E). Enzyme lysis experiments showed that the R-biofilms contained large amounts of protein, since proteinase K (100 μg/ml) at 37°C could completely disrupt these structures. However, Benzonase nuclease could not disrupt the structures (Fig. 2F). It may be that DNAs in the R-biofilm are protected from nuclease digestion by protein structures. Proteinase lysates generated from one classic R-biofilm with a diameter of 5 mm, which was formed after 24 h of incubation in a test tube, were plated onto LB agar plates and cultured at 37°C overnight. About 1 × 10^3^ CFU of bacteria were counted after 10,000-fold dilution (Fig. 2F). That is, there were approximately 1 × 10^7^ CFU of viable bacteria in each R-biofilm. To investigate the ability of R-biofilms to protect bacteria, we treated strain 355 R-biofilms (the discoid form at 24 h) of similar sizes with different bactericidal agents, including 75% ethanol for 20 min, 10% hydrogen peroxide for 60 min, UV radiation for 5 min, and an autoclave sterilizer at 121°C for 30 min. After treatments, the R-biofilms were injected intraperitoneally into mice (5 mice per group), and the mice were dissected 12 h later. The livers of the mice in the groups other than that in which the R-biofilms underwent autoclave sterilization were infected with strain 355. This suggests that R-biofilms are resistant to multiple common clinical bactericidal techniques and that they can effectively release bacteria within a host (Fig. 2G; see also Text S1).

10.1128/mSphere.00336-20.1 TEXT S1 Supplemental information, including the preliminary experiments on animal experiments, plasmid pB16Kp construction, bacterial strains, sources, etc. Download Text S1, PDF file, 0.1 MB. Copyright © 2020 Liu et al. 2020 Liu et al. This content is distributed under the terms of the Creative Commons Attribution 4.0 International license.

In summary, a novel type of biofilm (the R-biofilm) caused by DNA breaks is described in this report. R-biofilms are mainly composed of extracellular proteins and/or DNAs and might be built as a refuge for bacteria to overcome adverse circumstances. They can release a large number of bacteria when injected into the bodies of mice, resulting in infection. The bacterial SOS response system is most likely involved in the formation of R-biofilm structures. The signaling molecules and the metabolic pathways participating in this process should be clarified next. In addition, there are open questions about this discovery. Do any other bacteria form R-biofilms when their DNA undergoes breaks? Could R-biofilms be produced in medical environments (such as in indwelling urinary catheters) or the intestines of patients? Is any capsular polysaccharide involved in the construction of R-biofilms? And could we use R-biofilms as a functional biological material, given their good ductility?
