# Supplementary figures and images for: Nonclassical Biofilms Induced by DNA Breaks in Klebsiella pneumoniae
Source: mSphere. 2020 Jun 10;5(3):e00336-20. doi: 10.1128/mSphere.00336-20 (PMC7289706; doi:10.1128/mSphere.00336-20)

**FIG S1**

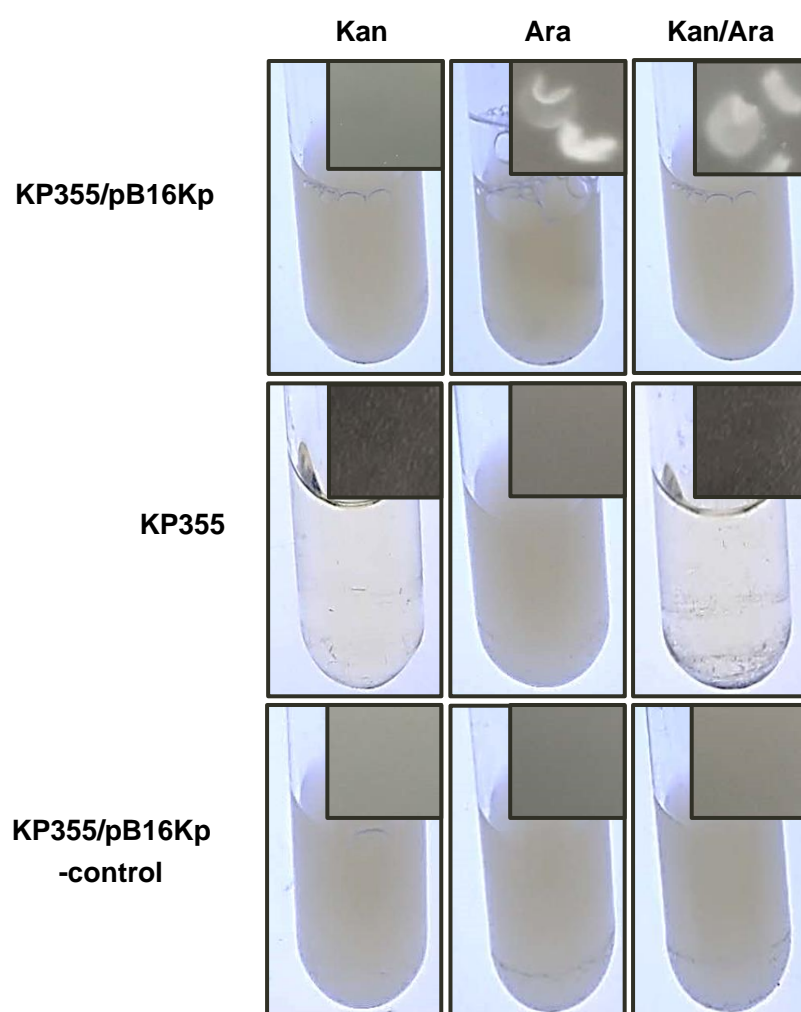

Supplement: FIG S1 [file mSphere.00336-20-sf001.pdf]

FIG S2

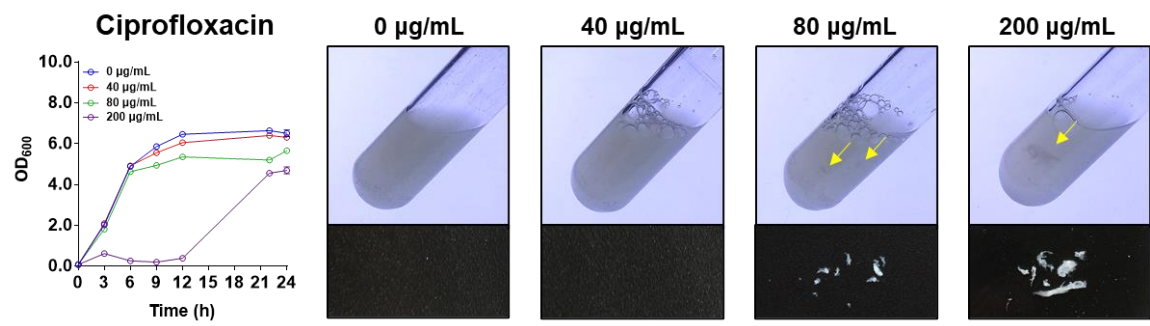

Supplement: FIG S2 [file mSphere.00336-20-sf002.pdf]

FIG S3

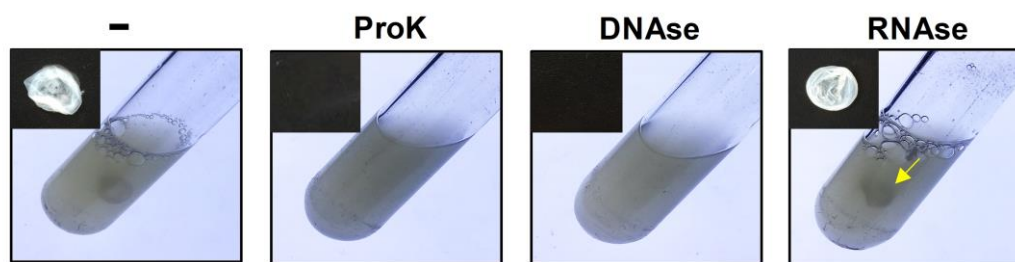

Supplement: FIG S3 [file mSphere.00336-20-sf003.pdf]

FIG S4

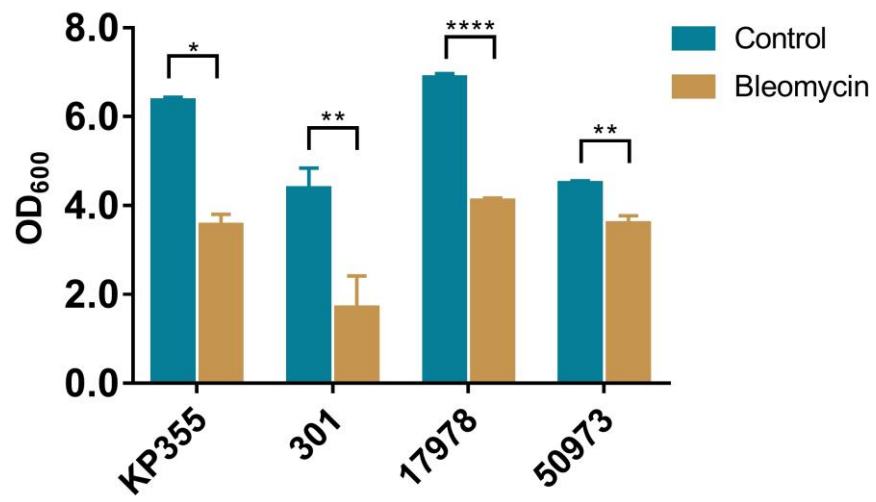

Supplement: FIG S4 [file mSphere.00336-20-sf004.pdf]

FIG S5

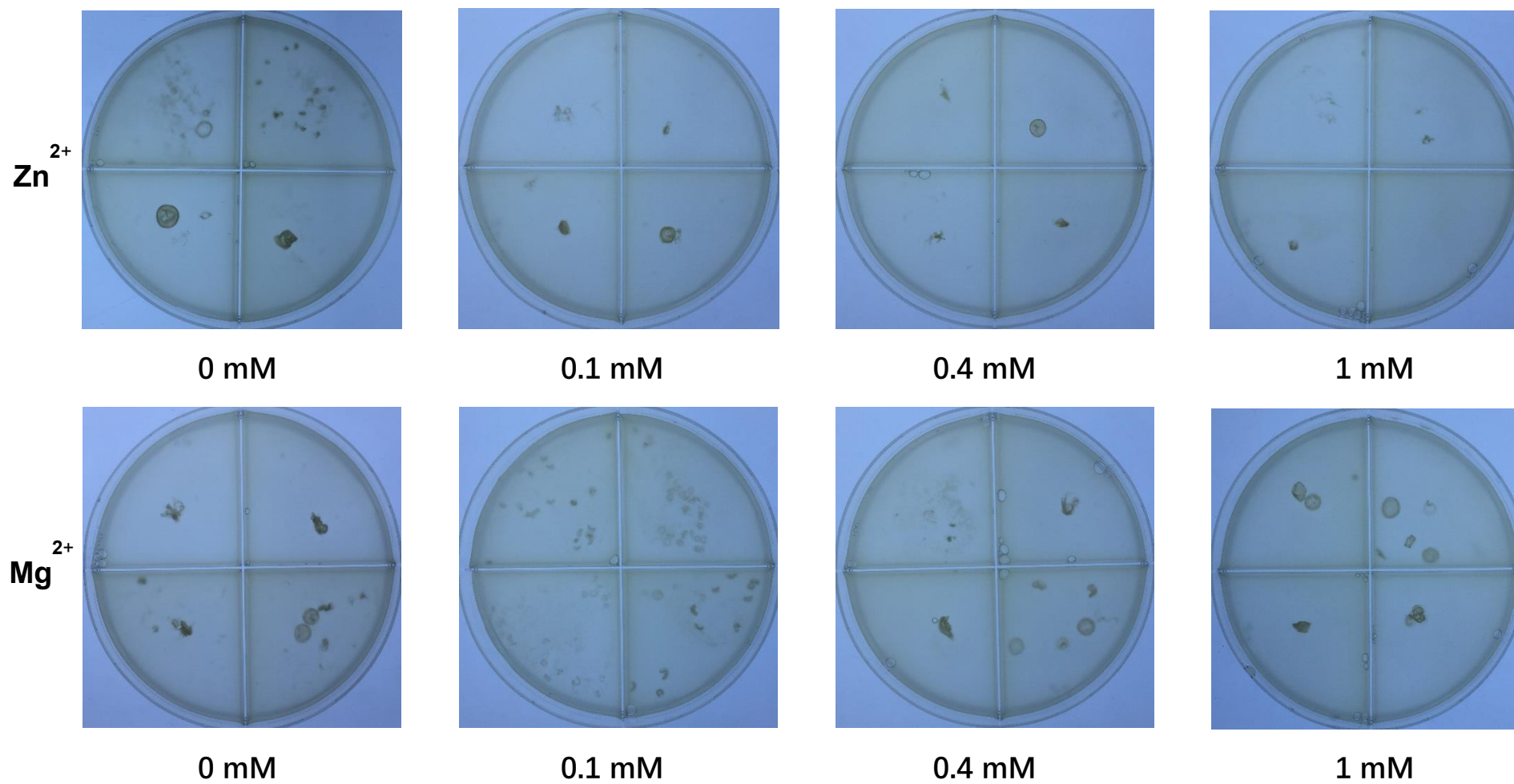

Supplement: FIG S5 [file mSphere.00336-20-sf005.pdf]

FIG S6

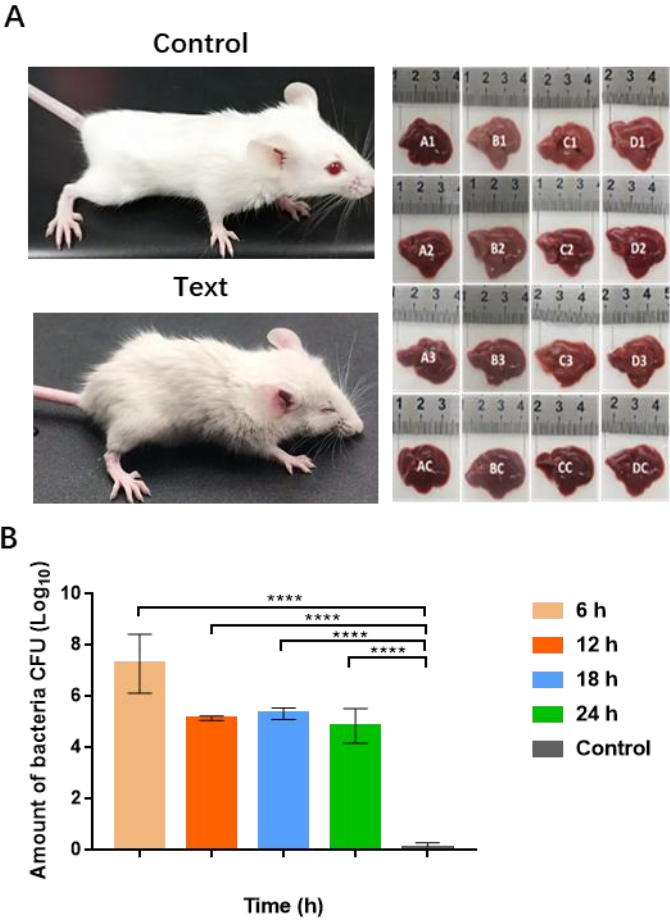

Supplement: FIG S6 [file mSphere.00336-20-sf006.pdf]

FIG S7

A

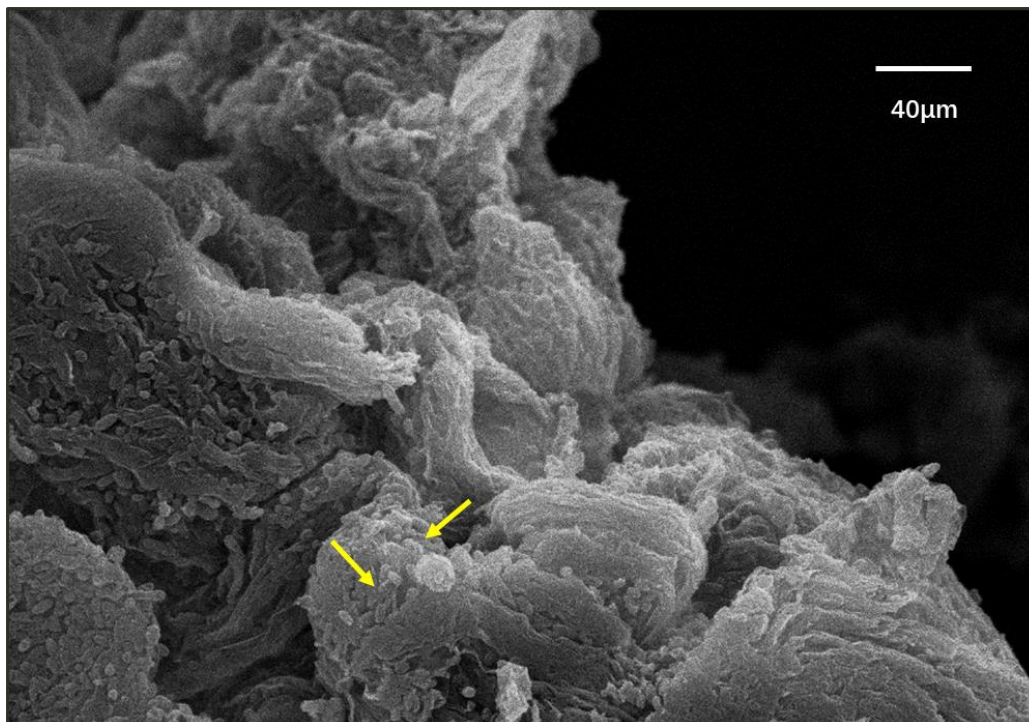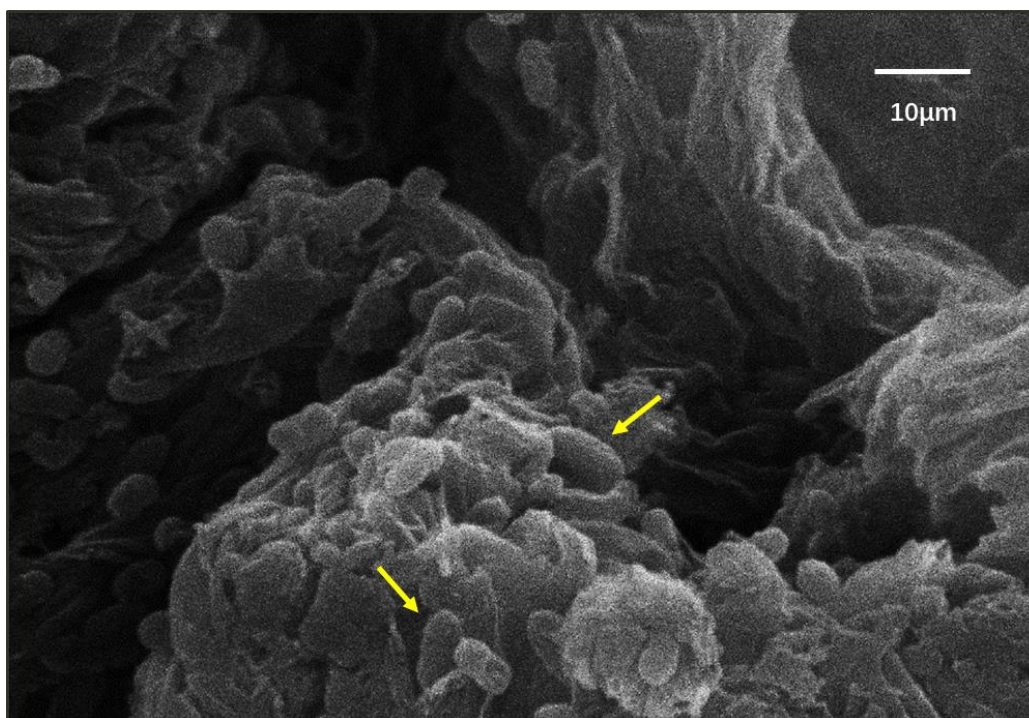

B

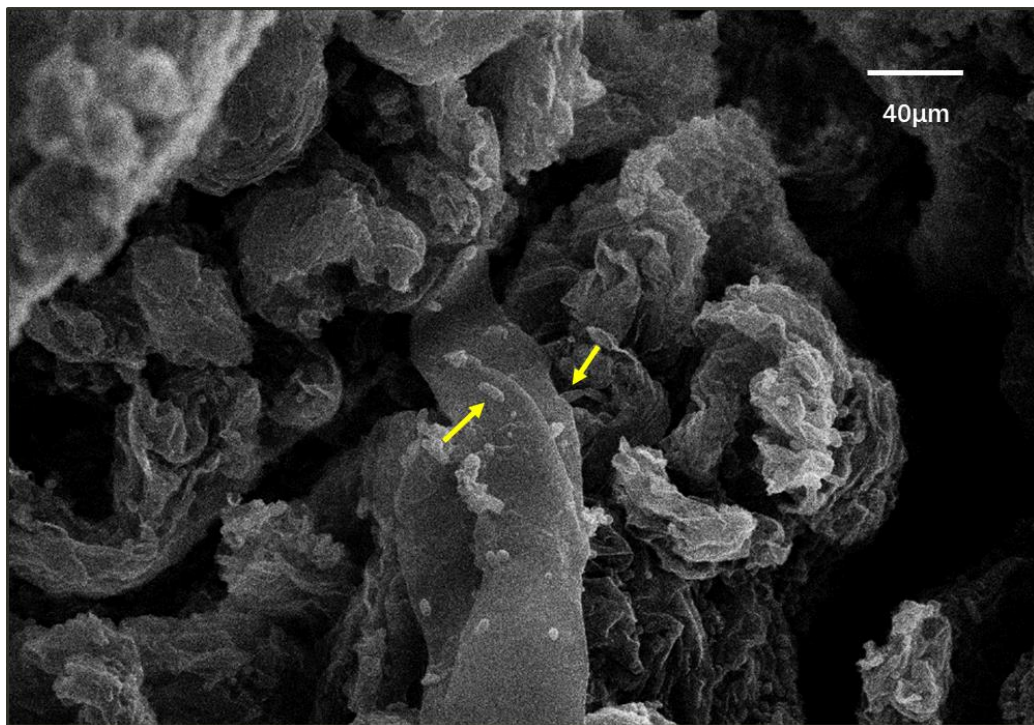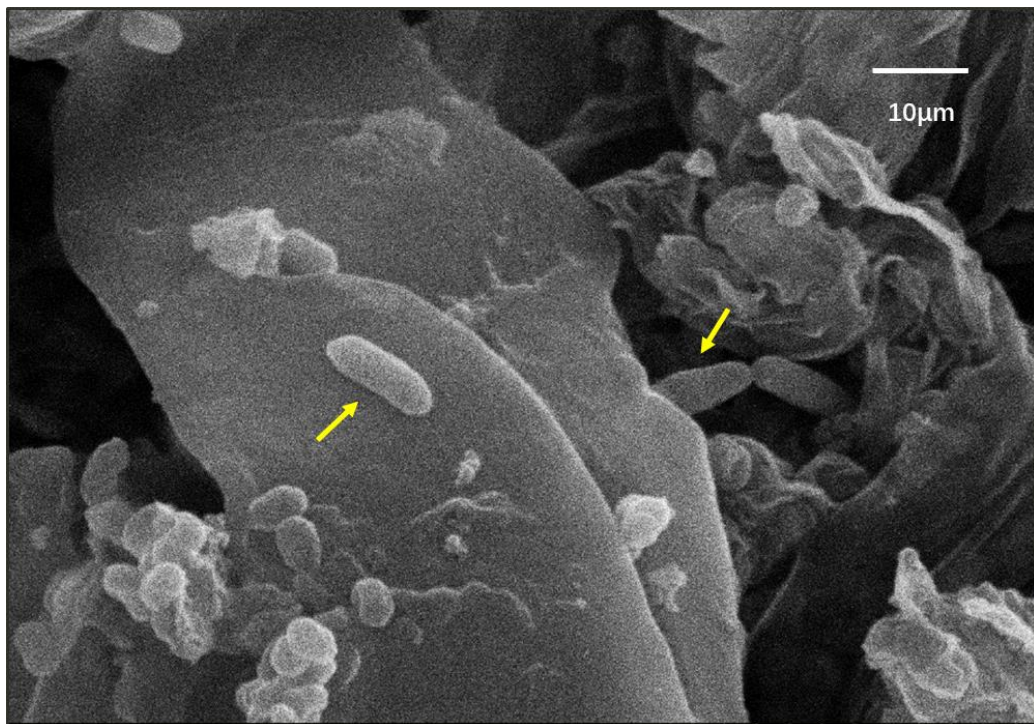

Supplement: FIG S7 [file mSphere.00336-20-sf007.pdf]
