# Supplementary material for: Nonclassical Biofilms Induced by DNA Breaks in Klebsiella pneumoniae
Source: mSphere. 2020 Jun 10;5(3):e00336-20. doi: 10.1128/mSphere.00336-20 (PMC7289706; doi:10.1128/mSphere.00336-20)
Supplement: TEXT S1 [file mSphere.00336-20-s0001.pdf]

Text S1

Supplemental information:

To determine the effect of mouse dissection time on the number of bacteria in the liver, we intraperitoneally injected a solution containing 2.5×10^{7} CFU of bacteria into four groups of mice and harvested their livers after 6, 12, 18, and 24 h, respectively. In each group, three mice were injected with bacteria and one with saline (control). There were no significant differences in liver weight with regards to the time of dissection. The number of bacteria was slightly higher in the livers harvested at 6 h, and similar between the livers harvested at the other time points (Fig. S6). Therefore, dissection times <30 h did not significantly affect the number of bacteria in the liver. As such, we chose to harvest the livers 12 h after injection in all subsequent experiments.

To determine the number of viable bacteria within R-biofilms, we selected three R-biofilms of similar morphology and used proteinase K for enzymolysis prior to diluting, swabbing, and counting the colonies. The mean number of viable bacteria within the R-biofilms was 2.5×10^{7} CFU.

Plasmids: Plasmid pB16Kp construction: we used pBBR1MCS2 as the backbone, which included a replication protein (*rep*), origin of replication (*oriV*), junction-transfer-inducing gene (*mob*), and kanamycin resistance gene (*kan*). The arabinose operon gene *araC*, Cas9 gene, and gRNA gene (the promoter: 5′-ttgacaattaatcatccggctcgtataatg-3′, and N20 were constructed on primers) were amplified by PCR and then connected to the plasmid backbone by the Goldengate

method from 5′- to 3′-. *araC*, the Cas9 gene, and the gRNA gene described above were amplified from pKD46, the Cas9 expression plasmid (Addgene reference number: 42876), and pTargetF (Addgene reference number: 62226), respectively.

Bacterial strains: *Klebsiella pneumoniae* strains 348, 350, 352, 353, 354, and 355 were isolated at Sir Run Run Shaw Hospital of Zhejiang University.

Animals: All animals were purchased from the Beijing Vital River Laboratory Animal Technology Co., Ltd and were also housed in the Center, which had a constant ambient temperature (23 ± 3 °C) and humidity (55 ± 5%). Food, bedding, and water were changed every 4 days. All animal experiments were approved by and performed in accordance with the recommendations of the Academy of Military Medical Sciences Institutional Animal Care and Use Committee.
